# What Is the Role of Plant-Based Alternatives to Animal Foods in the Great Food Transformation: A Narrative Review

**DOI:** 10.1016/j.tjnut.2025.101275

**Published:** 2025-12-29

**Authors:** Beatriz Philippi Rosane, Linda Okoren, Barbara Vad Andersen, Derek V Byrne, Susanne Gjedsted Bügel

**Affiliations:** 1 Department of Nutrition, Exercise and Sports, University of Copenhagen, Frederiksberg, Denmark; 2 Sino-Danish College, University of Chinese Academy of Sciences, Beijing, China; 3 Food Quality Perception and Society Team, iSense Lab, Department of Food Science, Faculty of Technical Sciences, Aarhus University, Aarhus, Denmark

**Keywords:** plant-based alternatives, animal foods, plant-based meats, plant-based milk, great food transformation

## Abstract

Several plant-based alternatives to animal foods (PBAFs) have been introduced to the market in the past decade to provide consumers with an easy substitution for animal protein. Despite the extensive literature on the nutritional composition of these plant-based products, these products must be assessed considering their impacts on diets, the environment, and society. This critical review aims to evaluate the role of plant-based alternatives to animal foods in the Great Food Transformation and their potential health effects. Results of 9 diet modeling studies have indicated that substituting animal foods with plant-based alternatives would put certain populations at risk of inadequate intake of micronutrients, particularly of bioavailable iron, vitamin B12, calcium, and iodine. Nutritional composition studies have shown that meat alternatives are generally similar to meat in protein and micronutrient content but with higher carbohydrates and sodium. Milk and yoghurt alternatives made from cereals, coconut, and nuts have significantly lower protein levels compared with their animal counterparts, whereas soy-based alternatives are the most comparable to animal dairy products. This review shows that there are nutritional pitfalls from the substitution of animal foods with PBAFs. There is a window of opportunity to use these industrially produced foods to improve nutrition. This review concluded that PBAFs could be used to ease the Great Food Transformation by providing foods that resemble function and appearance to consumers, by offering familiar tastes and dishes. Moreover, PBAFs should be considered in future nutritional policies.

## Introduction

The Great Food Transformation is the term used by the EAT-Lancet Commission [1,2] to refer to a positive change toward more sustainable and healthy diets. The Planetary Health Diet proposed by the EAT-Lancet Commission [1], is plant-rich, mainly based on cereals and legumes, with small amounts of animal proteins.

Concurrently, consumers have become more aware of the urgent need to switch their diets to more environmentally friendly eating patterns [3,4]. In this context, the food industry has released a wide variety of plant-based foods that mimic the appearance and functionality of animal-based foods [3,5]. A justification for producing plant-based foods that mimic the appearance, taste, and function of animal protein are to ease the transition toward more sustainable diets by allowing consumers to maintain their habitual meal structures and current cooking skills with easy “like-for-like” replacements [5].

These plant-based alternatives to animal foods (PBAFs) have been analyzed in different studies in the past decade, including nutritional composition [[6], [7], [8], [9]], environmental impact [[10], [11], [12]], consumer acceptability and perceptions [[13], [14], [15]]. However, these aspects have not been considered together in a comprehensive analysis.

For example, de las Heras-Delgado et al. [6] found that most PBAFs in Spain are lower in protein and higher in sugar, fiber and sodium than animal counterparts. Using Nutri-score, the same study found that meat PBAFs are healthier than meats, but cheese PBAFs are less healthy than dairy cheese [6]. Similar, Romão et al. [8] found that PBAFs for dairy and eggs in Brazil were higher in carbohydrates and fiber, whereas only cheese and milk PBAFs are lower in protein than dairy.

Environmental impact assessments of PBAFs have shown that PBAFs have much lower use of natural resources and carbon footprint than their animal counterparts when comparing similar serving sizes. The study of Kustar and Palatino-Echeverri [12], found that even highly processed PBAFs, such as plant-based burgers, have much lower (between 87% and 96%) land use, water use and greenhouse gas emissions than beef patty.

Consumer studies show that, even though PBAFs are getting more popular, consumers are not totally satisfied with these products. Particularly, PBAFs poor sensory properties and high prices are major barriers for PBAFs consumption [13]. Most recently, diet modeling studies have also considered the theoretical implications of substituting animal foods with PBAFs [[16], [17], [18], [19], [20]]. These studies show a risk of insufficient micronutrient intake if a total or large percentage of animal proteins are substituted with PBAFs currently available for purchase. Risk changes according to the populations’ age and geographical location, but most studies cite potential insufficient intake of complex B vitamins and calcium [[16], [17], [18], [19], [20]]. These studies focus mainly on nutritional pitfalls, without discussing other aspects of sustainability.

Considering that sustainable healthy diets should not only be good for health and environmental well-being but also safe, equitable, affordable, accessible, and culturally acceptable [21], a more holistic approach is needed when judging the quality of the PBAFs. However, considering the variety of alternatives and the complexity of adopting a comprehensive, holistic approach, this review focuses primarily on the nutritional composition but also touch on other aspects of sustainability (i.e., environmental impact and consumer perception). This review contributes to the existing literature by discussing the role of PBAFs to animal foods that mimic the appearance and functionality of said animal counterparts in the Great Food Transformation and investigates if PBAFs are appropriate replacements for animal foods in a transition toward more sustainable diets in terms of nutrition and other sustainability aspects.

## Methods

This study is a critical review based on a narrative approach. Relevant literature was obtained from a search in scientific databases (i.e., PubMed and Web of Science) and an artificial intelligence online platform for academic papers search Elicit [22]. When searching for plant-based foods studies, they were only considered for this review if they investigated plant-based foods that mimic the appearance and functionality of animal foods, such as plant-based “meats” (sausages, burgers, nuggets, patties, fillets, and similar), plant-based drinks (PBDs) or “milks” and plant-based “eggs” or similar. We focused our search on nutritional composition, environmental impact assessments, and consumer perception.

Additional studies were included by citation tracking. Only studies with products available for consumption were included, excluding those with new products or theoretical recipes.

For the nutritional aspect, 2 different types of studies were reviewed. First, modeling diet studies were analyzed. Diet modeling is a mathematical approach to test the effects of dietary changes based on hypotheses, consumer projections or application of new recommendations [23,24]. These studies have investigated changes in nutritional intake in scenarios of partial or total substitution of animal foods with PBAFs.

Second, studies that investigate the nutritional composition of PBAFs were reviewed. The results were analyzed differently depending on the product category. For PBAFs of meat, yoghurt and cheese, a large variety of animal and PBAF products was observed. This is because these categories include several foods within each. Using meats as an example, the variety starts by animal origin (e.g., poultry, cattle, pork, fish) and processing or cut (e.g., minced beef, bacon, sausage, nuggets, chicken breast, patty). Therefore, the main findings of these studies have been summarized, focusing on the comparison between animal and plant-based products presented in the studies reviewed [6,8,9,18,[25], [26], [27], [28], [29], [30], [31], [32], [33], [34]]. Additionally, we extracted data from nutritional composition from the studies for all products, both animal and PBAFs. For meats, some studies did not include information about the animal counterpart. In such cases, data were sourced from the USDA database. For PBDs, or milk PBAFs, only products with a single raw material and not flavored were considered for the present study's analysis. The mean and SD values of nutrients were calculated using Microsoft Excel Version 2501. Often, mean values were considered due to data availability and, therefore, no statistical analysis on differences between animal and PBAFs was performed. The collected data are available in Supplemental Material 1.

**TABLE 1:** Summary of scenarios and main findings of diet modeling studies with plant-based alternative foods

Study	Population	Scenarios	Main findings
Farsi et al. [16] (2022)	United Kingdom population (ages between 1.5 and 95 y)	Model 1: replacement of individuals’ meat intake in 25%, 50%, 75% and 100% with meat alternatives^1^ Model 2: comparison of 100% meat intake replacement by different ingredients [i.e., mycoprotein, legumes (bean and pea), tofu, nut, soy] Model 3: comparison of 100% meat intake replacement by fortified and unfortified products	Model 1: linear decrease of protein and saturated fats with an increase of meat alternatives; a significant increase in fiber and sodium. No significant changes in iron or vit B12. Model 2: the highest increase in fiber was seen for substitution with nuts, followed by vegetable and mycoprotein products, whereas the lowest was seen for tofu. The highest reduction of saturated fats in the nut’s product scenario, whereas the biggest reduction of protein would be for nuts, vegetables and legumes. Intake of vitamin B12 would be significantly lower with tofu, nuts, and soy products, whereas iron tofu products would increase the intake. Sodium would only not increase significantly with nut products. Model 3: fortified products were associated with lower protein decrease but higher sodium intake. The intake of iron would be significantly higher with fortified products and would be within United Kingdom dietary recommendations and comparable current intake. In the unfortified scenario, vitamin B12 would be significantly lower but still within recommended values.
Lawrence et al. [17] (2023)	Australian population (ages between 2 and 90 y)	Total substitution of “Easily Swappable Animal Source Meat” (39.5% beef, 38.1% chicken, and 22.4% sausages) and fluid cow milk for plant-based alternatives^2^ considering projections of intake of plant-based meat and dairy for 2030 Accelerated meat scenario (∼42 g/person/day); conservative meat scenario (∼6.5 g/person/d); accelerated milk scenario (100% substitution); conservative milk scenario (∼1 L per person per week being substituted)	Accelerated meat and milk scenario: the most pronounced decline in nutrient intake was of iodine (19%) and vitamin B12 (19%), and increased intake of iron (15%), sodium (7%) and magnesium (5%). Decline in intake of phosphorus, riboflavin, niacin and n-3 fatty acids of 6%–8%. Conservative meat and milk scenario: the decline in nutrient intake was small (<5%).
Marchese et al. [18] (2024)	Australian population (≥19 y.o.)	Model 1: 50% replacement of meat and dairy by traditional alternatives^3^ for meat and dairy, and plant-based drinks Model 2: 100% replacement of meat and dairy with traditional alternatives for meat and dairy and plant-based drinks Model 3: 50% replacement of meat and dairy with traditional alternatives, meat and dairy analogs Model 4: 100% replacement of meat and dairy with traditional alternatives, meat and dairy analogs	The diet quality index (DGI)^4^ increased for all models across all sex and age groups compared with baseline and better diet qualities were seen on model 2. Similar DGI scores were observed for models 1 and 3. Model 4 resulted in the lowest increase in DGI. All models resulted in a decreased intake of protein, fats, and sugars. For protein, modeled values were still higher than EAR. The highest decrease in protein and fats was seen for model 2. On total substitutions (models 2 and 4), the modeled intake of n-3 fatty acids was below AI for all sex and age groups. Carbohydrates and fiber increase in all models, with the greatest increases in models 2 and 4. Folate increased in all models, whereas vitamin B12 was lower in traditional models (1 and 2) but still above EAR. For models 3 and 4, there was an increase of vitamin B12 for young males and females (≥19 to 30 y).
Salomé et al., 2021 [19] (2021)	French adults (18–79 y.o.)	Meat substitution model: tested the substitution of all meats with 56 plant-based substitutes Milk substitution model: tested the substitution of all milk with 16 plant-based substitutes Dairy dessert substitution model: tested the substitution of all dairy desserts and yoghurt with 24 plant-based substitutes	Increased risk of deficiency Meat substitution model: riboflavin, vitamin B12 and bioavailable iron Milk substitution model: calcium (unfortified products), vit B12, riboflavin, iodine and potassium Dairy dessert substitution model: calcium (unfortified products), riboflavin, and iodine Decreased risk of deficiency Meat substitution model: folate and calcium Milk and dairy dessert substitution models: linoleic and α-linolenic acids, fiber and bioavailable iron
Seves et al., 2017 [20] (2017)	Dutch adults (19–69 y.o.)	No meat and dairy:100% substitution Less meat and dairy: partial substitution Only foods fully or predominantly consisting of meat or dairy with plant-based alternatives (fish not included). Meats were substituted with vegetarian meat replacements, soy products or pulses.	No meat and dairy: decreased intake of saturated fats (35%–36%), calcium (25%), zinc (45% for men and 29% for females), and vitamin B12 (41% for men and 27% for females). Increased intake of mono- and disaccharides (16%–18%), iron (21% for men and 19% for females), vitamin D (32%–34%) Less meat and dairy: decreased intake of zinc (10%–12%). Increased intake of iron (7% for men and 6% for females) Both scenarios: increased intake of fiber but still bellow AI.
Taeger and Thiele[108] (2024)	German adult population (25–49 y.o.)	Linear programming modeling to compare 3 dietary scenarios: 1. Diets with milk and dairy products 2. Diets with nonfortified soy-based alternatives 3. Diets with soy-based alternatives fortified with calcium, vitamins D, B2, and B12	Consuming nonfortified soy alternatives can lead to calcium and vitamin B12 deficiencies, which can only be avoided by making significant changes to the diet. Consuming fortified soy-based alternatives can adequately replace the nutrients found in milk and dairy products.
Tso and Forde [33] (2021)	United States adult males (20–49 y.o.)	Modeling of 2 versions (traditional and novel, that is, with PBAF) of flexitarian, vegetarian, and vegan diets.	All scenarios were lower in cholesterol, vitamin B12, and zinc (compared with an omnivorous diet). The models with PBAF were excessive in saturated fats, sodium, and sugar. They were also lower in calcium, potassium, magnesium, zinc, and vitamin B12 than the daily requirements. The lower micronutrient intake was explained by the PBAFs being lower in micronutrient content than their animal counterparts. The vegetarian and vegan novel scenarios were only adequate in phosphorus and iron, whereas their traditional scenarios were adequate for all micronutrients, except for vitamin B12 and calcium in the vegan traditional scenario.
Wanders et al. [38] (2025)	Dutch adults (18–70 y.o.)	Different modeling scenarios based on meat replacement: 1. No protein replacement (simply omitting meat from the diet) 2. Replacement with PBAF currently available in the Dutch market 3. PBAF in equal protein content 4. PBAF with low digestibility (assuming that PBAF have 75% protein digestibility, like legumes) 5. Source-specific scenarios	No protein replacement had 85% protein adequacy; all other scenarios had >97% adequacy Utilizable protein in the different scenarios was 50% for no protein replacement, 81% in PBAF with low digestibility, 86% in the PBAF currently available and 90% in the equal protein content. For source-specific scenarios utilizable protein varied from 67% to 96%. Protein quality was mostly impacted by the total protein content of PBAFs, but lysine content and protein digestibility also played a role.
Zhang et al. [107] (2020)	Australian adolescents (12–18 y.o.) and older females (>50 y.o.)	Adolescent scenario: modeling intake of 3.5 servings of cow milk and plant-based drinks based on tree nuts/seeds, legumes, grains, coconut and mixed alternatives. Older females scenario: modeling intake of 4 servings of cow milk and plant-based drinks based on tree nuts/seeds, legumes, grains, coconut, and mixed alternatives. One serving is equivalent to 250 mL. The analysis focused on protein, sugars, calcium, and zinc.	The modeling analysis suggested that cow milk is a significant source of protein, calcium and zinc in adolescents and older females, providing 50% or over of the daily recommended intake in both population groups. Adolescents: legumes are the most comparable plant-based alternative to cow milk in terms of protein and calcium. Other plant-based milks are less adequate for protein and zinc. Older females: legume and grain-based plant milks can meet calcium needs similarly to cow milk. Protein and zinc intake may be lower with plant-based alternatives, requiring careful selection of fortified products or dietary adjustments.

1 Meat alternatives were based on the share of meat alternatives in the United Kingdom market (vegetable 23%, mycoprotein 18%, bean 17%, soy 15%, nut 14% and tofu 13%).

2 The substitution of milk was calculated as being 47.6% from soy drinks, 44.2% from almond drinks and 8.2% from “others,” following Australian market information.

3 Traditional alternatives: legumes, beans, and tofu.

4 DGI stands for dietary guidelines index, based on the Australian Dietary Guidelines for adults.

**TABLE 2:** Nutritional composition of meat products divided by food product and origin (animal and plant-based)

Food product	Origin	*N* of product^1^	Energy (kcal)	Carbohydrates (g)	Protein (g)	Total fats (g)	Saturated fats (g)	Cholesterol (mg)	Dietary fiber (g)	Sodium (mg)	Iron (mg)	Zinc (mg)	Vitamin B12 (μg)	Vitamin B2 (mg)
Bacon^2^; Bryngelsson et al. [29] (2022), Tso and Forde [33] (2021)	Animal	1	393	0	13.7	37.1	12.6	66	0	751	0.38	1.14	0.5	0.08
	Plant-based	6	308 ± 181	4.52 ± 0.67	8.44 ± 0	29.8 ± 23.8	12.9 ± 16.2	0	4.39 ± 3.54	2132	1.94 ± 0.23	0.33	0.19 ± 0.27	—
Beef patties/burgers^2^; Tso et al. [9] (2020), Alessandrini et al. [25] (2021), Cole et al. [26] (2022), Romão et al. [28]; (2022), Bryngelsson et al. [29] (2022), van Vliet et al. [32] (2020)	Animal	68	276 ± 30	0.22 ± 0.31	20.2 ± 2.83	21.1 ± 3.59	8.53 ± 1.15	76.0 ± 11.3	0.06 ± 0.49	109 ± 148	2.17 ± 0.35	4.84	2.65	0.18
	Plant-based	144	215 ± 73	16.3 ± 12.7	15.1 ± 8.7	15.1 ± 8.68	3.25 ± 3.96	0.04 ± 0.10	5.21 ± 4.23	443 ± 210	3.41 ± 0.37	2.43 ± 3.44	1.33 ± 1.88	0.18 ± 0.25
Beef jerky^2^; Tso and Forde [33] (2021)	Animal	1	410	11.0	33.2	25.6	10.8	48.0	1.8	1780	5.42	8.11	0.14	—
	Plant-based	1	321	28.6	35.7	3.57	0.0	0.0	0.0	2286	4.29	1.97	—	—
Breaded chicken^2^; Alessandrini et al. [25] (2021), Romão et al. [28] (2022)	Animal	40	252 ± 12	14.9 ± 0.07	14.3 ± 0.61	14.5 ± 1.17	2.77 ± 0.43	38.5 ± 3.54	1.33 ± 0.32	448 ± 145	0.98 ± 0.19	0.70 ± 0.12	0.23 ± 0.06	0.08 ± 0.02
	Plant-based	35	218 ± 62	16.9 ± 10.0	10.0 ± 12.9	11.2 ± 6.07	1.33 ± 1.23	—	4.26 ± 1.85	509 ± 191	—	—	—	—
Canned fish^2^; Romão et al. [28] (2022)	Animal	3	150 ± 62	0.03 ± 0.05	22.3 ± 2.92	6.23 ± 5.26	1.20 ± 0.86	89.0 ± 74.9	0.00 ± 0.00	302 ± 80	2.21 ± 0.64	1.00 ± 0.33	6.15 ± 3.26	0.17 ± 0.08
	Plant-based	8	194 ± 88	14.7 ± 5.68	8.75 ± 4.64	11.0 ± 7.07	2.63 ± 1.56	—	6.41 ± 5.58	416 ± 187	—	—	—	—
Chicken fillet/tenders; Tso et al. [9] (2020), Alessandrini et al. [25] (2021), Romão et al. [28] (2022), Tso and Forde [33] (2021)	Animal	69	117 ± 42	1.40	18.8 ± 6.43	3.86 ± 1.64	1.55 ± 0.21	11.0	0.10 ± 0.14	425 ± 318	2.9	0.3	—	—
	Plant-based	52	172 ± 51	8.53 ± 5.41	18.6 ± 10.9	6.64 ± 4.57	1.59 ± 2.88	47.5 ± 67.2	4.65 ± 4.88	481 ± 293	1.93 ± 1.27	0.77 ± 0.14	0.09 ± 0.12	—
Chicken nuggets/burger^2^; McClements et al. [27] (2023), Romão et al. [28] (2022)	Animal	2	281 ± 28	16.5 ± 0.35	15.0 ± 0.85	17.1 ± 2.47	3.91 ± 0.76	39.0 ± 7.07	0.35 ± 0.49	522	0.72 ± 1.01	0.75	0.23	0.06
	Plant-based	7	202 ± 64	12.8 ± 6.81	18.5 ± 14.2	8.94 ± 5.34	3.05 ± 4.33	0.00 ± 0.00	5.79 ± 6.49	392 ± 182	2.30 ± 0.07	—	—	—
Cold cuts^2^; Romão et al. [28] (2022), Bryngelsson et al. [29] (2022)	Animal	3	311 ± 36	2.31 ± 0.99	16.3 ± 2.53	25.3 ± 4.27	9.02 ± 1.84	75.7 ± 13.1	0.28 ± 0.55	772 ± 97	1.35 ± 0.18	2.33 ± 0.17	1.06 ± 0.08	0.18 ± 0.06
	Plant-based	22	191 ± 44	8.33 ± 5.90	14.2 ± 3.36	10.3 ± 4.32	1.69 ± 1.86	—	4.67 ± 4.68	528 ± 188	2.75	—	1.25	0.23
Meatballs^2^; Alessandrini et al. [25] (2021), Cole et al. [26] (2022), Romão et al. [28] (2022)	Animal	9	253 ± 47	8.06	17.8 ± 4.88	17.1 ± 7.14	6.12 ± 2.14	—	1.55 ± 1.06	493 ± 245	1.77	1.66	1.0	0.23
	Plant-based	38	151 ± 69	7.14 ± 6.35	15.7 ± 10.3	6.21 ± 5.02	6.61 ± 21.6	—	5.1 ± 4.4	483 ± 190	2.1	—	0.38	—
Minced beef; Alessandrini et al. [25] (2021), Romão et al. [28] (2022), Bryngelsson et al. [29] (2022), van Vliet et al. [32] (2020)	Animal	15	189 ± 8	53.1	19.9 ± 0.53	11.9 ± 0.7	4.76 ± 0.48	53.1	0.80 ± 11.3	91	1.77	4.07	1.77	0.18
	Plant-based	40	191 ± 28	12.1 ± 12.6	15.1 ± 6.3	9.44 ± 3.42	2.98 ± 3.25	—	5.82 ± 4.33	475 ± 238	2.63	—	—	—
Fishes; de las Heras-Delgado et al. [6] (2023), Tso et al. [9] (2020), McClements and McClements [27] (2023)	Animal	188	146 ± 43	0.00	24.0 ± 6.33	5.12 ± 3.58	0.91 ± 0.81	58.2 ± 8.37	0.00 ± 0.00	269 ± 172	0.49 ± 0.06	0.51 ± 0.05	4.86 ± 0.18	—
	Plant-based	18	133 ± 26	19.5 ± 17.6	5.77 ± 3.53	4.33 ± 0.85	0.60 ± 0.14	0.00 ± 0.00	3.67 ± 2.52	693 ± 446	—	—	—	—
Fish cakes^2^; Tso et al. [9] (2020), Romão et al. [28] (2022)	Animal	2	139 ± 103	7.50 ± 4.10	11.8 ± 2.64	6.77 ± 8.30	1.97 ± 2.79	46.0 ± 19.8	0.65 ± 0.78	544 ± 162	0.61 ± 0.16	0.56	0.13	—
	Plant-based	4	194 ± 70	15.8 ± 9.4	9.46 ± 4.33	10.9 ± 7.63	1.46 ± 1.27	—	5.00 ± 3.08	486 ± 70	—	—	—	—
Sausages^2^; Alessandrini et al. [25] (2021), Romão et al. [28] (2022), Bryngelsson et al. [29] (2022),	Animal	75	176 ± 117	1.63 ± 1.39	17.4 ± 1.46	11.0 ± 12.5	3.97 ± 4.80	50.3 ± 8.14	0.00 ± 0.00	1095 ± 184	0.94 ± 0.42	1.82 ± 0.29	0.75 ± 0.64	0.20 ± 0.06
	Plant-based	84	240 ± 115	13.1 ± 17.6	19.6 ± 12.8	12.9 ± 6.84	0.99 ± 0.47	—	5.15 ± 3.07	818 ± 453	—	—	—	—

Values are mean ± SD.

1 Sum of all samples included. Note: only averages were available for sources 2, 4, 5, and 10.

2 USDA, Agricultural Research Service, n.d.

## Results

On the basis of the literature search, the results were separated by study aim. First, an overview of modeling studies with different scenarios of substituting animal foods with PBAFs is presented. Second, results of nutritional composition studies of meat, milk, dairy, and egg PBAFs are shown.

### Diet modeling studies

Recently, different diet model studies have considered the nutritional impacts of changing toward more plant-rich diets [[35], [36], [37]]. Some studies have also considered the possibility of using PBAFs for partial or total substitution of animal foods; these studies were gathered for this review and are displayed in Table 1 [[16], [17], [18], [19], [20],^33^,^38^].

**TABLE 3:** Overview of nutritional composition of cow milk and plant-based drinks from almond, coconut, oat, pea, rice, and soy

Nutrients	Cow’s milk^1^^,^^2^^,^^3^^,^^4^^,^^5^^,^^6^^,^^7^	Almond drinks^2^^.^^3^^,^^4^^,^^5^^,^^6^^,^^7^^,^^8^^,^^9^^,^^10^^,^^11^^,^^12^^,^^13^^,^^14^^,^^15^^,^^16^^,^^17^^,^^18^^,^^19^^,^^20^	Coconut drinks^1^^,^^3^^,^^4^^,^^5^^,^^6^^,^^7^^,^^10^^,^^13^^,^^15^^,^^17^^,^^19^^,^^20^^,^^21^	Oat drinks^1^^,^^3^^,^^4^^,^^5^^,^^8^^,^^9^^,^^10^^,^^11^^,^^13^^,^^14^^,^^15^^,^^16^^,^^17^^,^^18^^,^^19^^,^^20^^,^^21^^,^^22^^,^^23^	Pea drinks^11^^,^^16^^,^^19^^,^^20^	Rice drinks^1^^,^^3^^,^^4^^,^^5^^,^^6^^,^^7^^,^^8^^,^^10^^,^^11^^,^^13^^,^^14^^,^^16^^,^^17^^,^^19^^,^^21^^,^^22^	Soy drinks^1^^,^^2^^,^^3^^,^^4^^,^^5^^,^^6^^,^^7^^,^^8^^,^^10^^,^^11^^,^^12^^,^^13^^,^^14^^,^^15^^,^^16^^,^^17^^,^^18^^,^^19^^,^^20^^,^^21^^,^^22^^,^^23^
N of products^24^	340	348	147	431	11	176	461
Energy (kcal)	94.1 ± 65.5	27.3 ± 10.6	41.7 ± 30.4	51.7 ± 16.6	38.9 ± 11.8	72.8 ± 79.6	48.7 ± 17.8
Carbohydrates (g)	4.8 ± 0.2	2.3 ± 1.9	3.4 ± 2.3	7.4 ± 1.8	2.2 ± 1.0	14.9 ± 18.1	3.7 ± 1.9
Sugars (g)^25^	2.7 ± 2.4	1.8 ± 1.7	1.9 ± 1.6	3.7 ± 1.6	1.9 ± 1.1	5.9 ± 2.1	2.4 ± 1.4
Dietary fiber (g)	0 ± 0	0.5 ± 0.4	0.3 ± 0.4	1.0 ± 0.7	1.5 ± 1.9	1.2 ± 1.9	0.6 ± 0.5
Total fats (g)	2.1 ± 1.2	1.8 ± 0.6	2.4 ± 1.6	1.7 ± 1.0	2.2 ± 0.7	0.9 ± 0.3	2.3 ± 1.6
Saturated fats (g)	1.2 ± 0.7	0.2 ± 0.1	2.1 ± 1.5	0.2 ± 0.1	0.8 ± 0.7	0.2 ± 0.2	0.4 ± 0.2
Protein (g)	3.3 ± 0.1	0.9 ± 0.7	0.5 ± 0.6	1.1 ± 0.6	2.2 ± 1.1	0.7 ± 1.6	3.2 ± 0.5
Calcium (mg)	117.3 ± 6.3	123.0 ± 62.8	99.1 ± 64.9	100.8 ± 52.8	126.7 ± 37.2	103.5 ± 45.5	109.4 ± 50.6
Sodium^26^ (mg)	45.3 ± 4.4	48.1 ± 33.2	47.1 ± 83.3	50.0 ± 54.7	66.0 ± 15.0	60.8 ± 61.7	54.8 ± 46.6
Iodine (mg)	9.6 ± 10.0	—	—	22.5	—	—	—
Vit B12 (mcg)	0.5 ± 0.1	0.3 ± 0.2	0.6 ± 0.5	0.2 ± 0.3	0.3 ± 0.1	0.4 ± 0.0	0.4 ± 0.2
Vit B2 (mg)	0.16 ± 0.01	0.1 ± 0.1	0.2	0.1 ± 0.0	0.2	0.1	0.2 ± 0.1

Values are mean ± SD.

1 Martínez-Padilla et al. [109] (2020).

2 Rodríguez-Martín et al. [110] (2023).

3 Singh-Povel et al. [31] (2022).

4 Smith et al. [111] (2022).

5 Walther et al. [113] (2022).

6 Chalupa-Krebzdak et al. [114] (2018).

7 Vanga and Raghavan [126] (2018).

8 Angelino et al. [112] (2020).

9 Kasapidou et al. [115] (2023).

10 Marchese et al. [18] (2024).

11 Romão et al. [8] (2022).

12 Scholz-Ahrens et al. [117] (2020).

13 J. Zhang et al. [116] (2023).

14 Moore et al. [118] (2024).

15 Pointke et al. [119] (2022).

16 Redan et al. [120] (2023).

17 Silva et al. [121] (2020).

18 Collard and McCormick [122] (2021).

19 Moshtaghian et al. [30] (2024).

20 Reyes-Jurado et al. [123] (2023).

21 Medici et al. [124] (2023).

22 Lo Turco et al. [34] (2023).

23 Siddiqui et al. [125] (2024).

24 Sum of all samples included. Note: only averages were available for sources 2, 3, 5, 7, 8, 10, 13, 19, 21, 22.

25 Not specified if those are added or naturally occurring sugars.

26 When only salt amounts were provided, sodium content was calculated considering that sodium is 40% of the atomic weight of salt chloride.

The reviewed modeling studies were calculated based on current dietary patterns in the Global North (i.e., Europe and Australia). Of the 9 studies selected, 4 have considered dietary changes caused by partial or total substitution of both milk and meats with PBAFs and/or “traditional” alternatives, that is, foods that have not necessarily been formulated as analogs to animal food, such as legumes, tofu, and tempeh. Additionally, 3 studies focused on total meat substitution with PBAFs, whereas 2 others focused on milk and dairy substitution with PBAFs.

In 4 of 9 studies reviewed, lower protein intake was observed in the PBAFs scenarios. One study focused on protein quality [38], and showed a decrease in protein bioavailability, but still adequate for the majority of the population. In their discussion, Wanders et al. [38] mention that protein content impacted protein adequacy more than other aspects.

In the reviewed modeling studies, 8 of 9 studies reviewed how a potential decrease in the intake of vitamin B12 occurs when meat and/or milk are substituted with PBAFs. Three of the 9 studies reviewed also mentioned a lower intake of zinc and riboflavin. Exclusively in the scenarios of substitution of milk with PBAFs, a lower intake of calcium (5 of 9 studies) and iodine (2 of 9 studies) is mentioned.

Two of 9 studies compared the calculated new intake with dietary recommendations and showed that, despite the decrease in intake, vitamin B12 would still be within the recommended intake for United Kingdom and Australian adults [16,18].

Contrastingly, 3 modeling studies with the substitution of meat have observed an increase in the total intake of iron with the use of PBAFs [16,17,20], whereas 1 study mentioned a decrease in the intake of bioavailable iron [19].

### Nutritional composition of PBAF

#### Alternatives to meat and meat products

Among the reviewed studies, a variation of results regarding the nutritional composition of PBAFs to meats was observed (see Table 2). This variation is likely due to diversity in the formulation of PBAFs and the different meat products and PBAF categories investigated. Regarding the results of studies comparing meats and their PBAFs, some commonalities were observed, as shown in Table 3 [6,9,[25], [26], [27], [28], [29],^32^,^39^,^40^]. First, when compared with animal foods, alternatives to meats were lower in total fats in 6 of 10 studies [6,[25], [26], [27], [28],^39^]. Namely, PBAFs were lower in saturated fats in 6 of 10 studies [9,[25], [26], [27], [28], [29]].

**TABLE 4:** Overview of comparisons of plant-based alternatives to meat products and their animal counterparts

Study	Method	Meat and PBAF comparison	Main findings
Alessandrini et al. [25] (2021)	Food label analysis of products available in supermarkets in the United Kingdom	Sausages (*n =* 72; PBAFs *n =* 41), burgers (*n =* 26; PBAFs *n =* 58), plain poultry (*n =* 68; PBAFs *n =* 20), breaded poultry (*n =* 38; PBAFs *n =* 22), mince (*n =* 14; PBAFs *n =* 16), and meatballs (*n =* 8, PBAFs *n =* 11)	Animal and PBAF were similar in energy. All animal products were higher in SFA and some in total fats than PBAF. All PBAFs had more fiber, and most had more salt than meat, whereas most PBAFs were lower in protein than their animal counterparts.
Bryngelsson et al. [29] (2022)	Food label analysis of products available for consumers in the Swedish retail market	Plant-based analogs to sausage (*n =* 31), bite/fillet (*n =* 20), burger (*n =* 19), cold cuts (*n =* 16), mince (*n =* 16), balls (*n =* 12), schnitzel (*n =* 11), nuggets (*n =* 7) and bacon (*n =* 5)	Compared with animal counterparts, PBAFs had lower saturated fats PBAFs were higher in carbohydrates, fiber, iron, folate and riboflavin No significant differences between PBAFs and animal food regarding energy, fats, protein, salt, and vitamin B12. However, nutritional quality changes between groups.
Cole et al. [26] (2022)	Food label analysis and data from the Mintel Global New Products Database of burgers available in the United States	Beef burger products (*n =* 41), plant-based imitations^1^ (*n =* 28) and veggie burgers^2^ (*n =* 89)	The study compares serving sizes, in which beef burgers were bigger (more grams) than PBAFs, more energy-dense and contained more fats, saturated fats, trans fats, cholesterol, and protein. Between the PBAF categories, plant-based imitations were more energy-dense and contained more fat, saturated fat, and protein than veggie burgers. Veggie burgers were higher in total carbohydrates and sugar. All PBAF contained more sodium, carbohydrates and fiber than beef burgers.
de las Heras-Delgado et al. [6] (2023)	Data extraction from the Spanish Veggie Base	Meat products (*n =* 432), unprocessed meat (*n =* 108) and meat alternatives (*n =* 432) (vegetable meatballs (*n =* 11), vegetable meat (*n =* 113), vegetable hamburgers (*n =* 155) and vegetable sausages (*n =* 39))	Most meat PBAFs were classified as A (42.8%) or B (21.5%) in the Nutri-Score (healthiest categories), similar to unprocessed meat (54.6% as A and 12.0% as B), whereas most meat products fell in the C (30.3%) and D (46.8%) categories. Using the NOVA classification, most meat alternatives were classified as processed foods (42.1%) and ultraprocessed foods (41.0%), whereas almost all meat products (94.7%) were ultraprocessed. Meat alternatives were more energy-dense and contained more sugar, salt, and fiber than unprocessed meat while being lower in total fats and protein.
McClements and McClements [27] (2023)	Review and data extraction from the USDA food database	Chicken nuggets (*n =* 1) and plant-based analogs (*n =* 2)	PBAFs were lower in energy, total and saturated fats, while higher in dietary fiber. Animal and PBAFs were similar in protein, total carbohydrate and sodium.
		Salmon (*n =* 1) and plant-based analogs to salmon (*n =* 2)	PBAFs were lower in protein and higher in sodium and calories. They also contain more fiber, iron, and calcium than salmon.
Nolden and Forde [39] (2023)	Review	Red meats and plant-based analogs	PBAFs are higher in fiber, Vitamin D, calcium and potassium, and lower in fats, and higher in sodium and sugars, and lower in iron, zinc and vitamin B12.
		Chicken and plant-based analogs	PBAFs are equivalent in protein quantity, higher in fat and have lower protein quality (essential amino acids).
Pingali et al. [40] (2023)	Food label analysis and data extraction from the USDA food database	Ground beef (*n =* 2; 80% and 90% lean) and plant-based burgers (*n =* 4; Beyond Meat, Impossible Foods, Dr. Praeger’s and Vivera)	PBAFs contain more carbohydrates and fiber than ground beef and are less caloric than 80% lean ground beef. 3 of the PBAFs contained more calcium and iron than ground beef, and all PBAFs contained more sodium than ground beef. Beyond meat and Impossible Food burgers contained similar or superior amounts of zinc and Vit B12 to ground beef.
Romão et al. [28] (2022)	Food label analysis of products in Brazilian supermarkets	Red meat and red meat products (hamburgers, minced beef, meatballs) and plant-based analogs Chicken breast, chicken hamburgers, and breaded chicken, and plant-based analogs Pork products (sausages and hams) Fish products (canned fish, i.e., tuna and sardines, and fish cakes)	Legumes (soy, peas, chickpeas, and beans) and gluten are used as sources of protein. All samples analyzed used ≥2 sources. Similar energy and protein quantity PBAFs were often higher in carbohydrates and dietary fiber. They have similar sodium content, except for minced beef and fish cakes (higher in PBAFs) and sausages (higher in animal products). PBAF presented similar or lower amounts of total and saturated fats. The most common food additives found were methylcellulose (58%), natural aroma (31.2%), and ascorbic acid (18.4%).
Tso et al. [9] (2020)	Label analysis and USDA food database	Chicken Breast (*n =* 1) and Mock Chicken^3^ (*n =* 1) Beef patty(*n =* 1), Impossible Burger patty^4^ (*n =* 1), Beyond Burger patty^5^ (*n =* 1) and black bean patty^6^ (*n =* 1)	Per serving, PBAFs are lower in calories and protein PBAFs are higher in fiber, but also carbohydrates, sugars, sodium The Impossible and Beyond burgers had a higher level of iron and similar total fat to beef patty. Mock Chicken, the Beyond burger, and the black bean patty had lower saturated fat content than their animal counterparts. Only animal foods had Vit B12 and zinc.
van Vliet et al. [32] (2020)	Review and label analysis	Ground beef (*n =* 1), soy analog (*n =* 1) and pea analog (*n =* 1)	The authors chose PBAFs with a similar macronutrient composition to ground beef. Plant protein isolates achieve the protein level, and thanks to fortification, micronutrients can be similar to or at higher levels than animal counterparts.

Abbreviations: PBAF, plant-based alternatives to animal food; TVP, texturized vegetable protein.

1 Plant-based imitations are plant-based products designed to mimic the taste, texture, and appearance of an animal food, in this case, a beef burger.

2 Veggie burgers are made to mimic the function of animal burgers but are not necessarily similar in taste, texture, or appearance. Source of plant-based protein: (1) soy, wheat, pea or TVP, (2) rice, soy, beans, wheat, mushroom, quinoa, millet or TVP, (3) gluten gluten-based, (4) soy soy-based, (5) pea protein-, rice protein- and mung bean protein-based, and (6) black beans and brown rice-based.

Second, 8 in 10 studies found that meat alternatives contain more carbohydrates than animal foods, including total carbohydrates [9,26,28,29,40], sugars [6,9], and dietary fiber [6,9,[25], [26], [27],^28^,^40^].

Third, a higher content of salt or sodium in the PBAF was reported in 5 of the 10 studies assessed [6,9,25,29,40].

Protein quantity also varied in the studies reviewed (TABLE 2, TABLE 3); protein quantity was lower in PBAFs in 5 studies [6,9,[25], [26], [27]]. The remaining studies did not observe differences in protein quantity between animal foods and PBAF.

The most significant difference came from micronutrient content. Namely, iron was higher in PBAFs in 3 studies [9,27,29], whereas lower in one. Similarly, vitamin B12 content was lower in PBAFs in 1 study [39], although similar or higher in a second study [40], and not present in PBAF in a third study [9].

#### Alternatives to milk and dairy products

##### Milks

Plant-based alternatives to milk or simply PBDs are often an emulsion of water and a cereal, nut, legume, seed, or pseudo-cereal [41]. PBDs demonstrate substantial variation in their nutritional composition (Table 4). Compared with cow milk, the biggest difference can be observed in protein and fat content. Soy drink is the closest to cow milk in protein content, followed by pea drink, whereas almond, oat, and rice milk contain significantly less protein. The fat content varies among PBDs, but its composition is generally higher in unsaturated fats compared with cow milk, apart from coconut drinks. Additionally, products based on oats and rice contain higher carbohydrate content than milk and other PBDs.

The micronutrient composition of PBDs exhibited considerable variability compared with cow milk, with the most notable differences observed in calcium and vitamin B12 content. Iodine content was largely undocumented, as only 1 study reported its content [30], whereas no data were available in the remaining studies.

##### Cheese

Plant-based cheese alternatives are primarily formulated from coconut oil, nuts, such as cashews and almonds, and soy, leading to considerable variation in nutritional composition. Most plant-based cheeses are significantly lower in protein than dairy cheese (Table 5) [6,8,30,[42], [43], [44], [45], [46], [47], [48], [49]]. Coconut-based cheeses (which are the most common ingredient used in this category) tend to be high in saturated fat and sodium, whereas nut-based cheeses generally contain lower levels of saturated fat and sodium (compared with dairy cheeses) [8,45,50]. Fortification rates for essential micronutrients are inconsistent, with Craig et al. [44], reporting only 19% of products containing added calcium, 14% including vitamin B12, and 1% incorporating vitamin D.

**TABLE 5:** Overview of studies comparing dairy products to plant-based alternatives

Study	Method	Dairy and PBAF comparison	Main findings
Alzahrani et al. [49] (2023)	Chemical analysis of iodine content of products collected in the United Kingdom	Cheese (*n =* 17; PB *n =* 11), milk (*n =* 25; PB *n =* 19), butter (*n =* 6; PB *n =* 4) and yoghurt (*n =* 14; PB *n =* 7) compared with PB alternatives	Iodine concentrations in dairy milk were 10 times higher than those in plant-based alternatives. Similar differences were observed in butter, yoghurt, and cheese. Although 20% of plant-based milk products were fortified with iodine, their iodine levels were still lower than those of equivalent dairy products.
Boukid et al. [42] (2021)	Food label analysis on the European market	Yoghurt (*n =* 86; PB *n =* 182) and cheese (*n =* 115; PB *n =* 114) compared with PB alternatives	Plant-based yoghurt alternatives were found to provide more energy, total fats, and carbohydrates than dairy yoghurt, whereas SFAs, sugars, and salt were similar. A larger proportion of plant-based products were labeled as low/no/reduced allergen compared with dairy products, catering to more consumer needs. Plant-based cheese alternatives showed more form versatility than dairy cheese and had higher total fats, SFAs, and carbohydrates, but lower protein, salt, and sugar.
Clegg et al. [43] (2021)	Food label analysis in the United Kingdom market	Milk (*n =* 51; PB *n =* 136), yoghurt (*n =* 78; PB *n =* 55), and cheese (*n =* 38; PB *n =* 109) compared with PB alternatives	Dairy milk contained more energy, saturated fat, carbohydrates, protein, and less fiber and free sugars than plant-based milk alternatives (*P* < 0.05). Dairy milk also contained more vitamin B2, vitamin B12, and iodine. Significant differences were found between yoghurt and cheese and their corresponding PBDAs in terms of energy, fat, saturated fat, carbohydrates, sugars, fiber, protein, salt, and calcium (*P* < 0.05). These differences affected nutrient intakes across different age groups, indicating that although PBDAs can be practical replacements for dairy products, they cannot be considered nutritional equivalents.
Craig et al. [44] (2022)	Food label analysis of products available in the United States	Plant-based cheeses (*n =* 245), no comparison to the nutritional value to dairy cheese	The study assessed cheese alternatives made from coconut oil, cashews, oats, almonds, soy, and other blends. Only 3% had 5 grams or more of protein. Fortification rates were 19% for calcium, 14% for vitamin B12, and 1% for vitamin D. Nearly 60% had high saturated fat levels, and 15% had low sodium levels. Cashew-based products had the highest protein and the lowest sodium and saturated fat levels, whereas coconut oil-based products had higher saturated fat and sodium and were often fortified with vitamin B12. Few products were good sources of protein or calcium.
Fresán and Rippin [45] (2021)	Food label analysis of products available on the Spanish market	Plant-based cheese (*n =* 40) compared with animal-based counterparts (*n =* 22)	Coconut oil-based cheese products made up most of the plant-based cheese alternatives and contained high saturated fat and salt content, with main ingredients being refined coconut oil and starches. The smaller groups of products, based on cashews and tofu, were lower in calories, fats, both total and saturated fat and salt compared with the dairy cheese. The dairy cheeses were higher in calories, total fats and protein than the plant-based cheese alternative and no significant differences were observed in the amount of saturated fats and salt between them.
de las Heras-Delgado et al. [6] (2023)	Spanish food composition database	Cheese (*n =* 80), milk and yoghurt (dairy products; *n =* 391) compared with PB alternatives (PB cheese *n =* 80; PB dairy products *n =* 391)	Plant-based cheeses were generally lower in protein and total fat but higher in salt/sodium compared with those of animal origin. Saturated fat content varied depending on the main ingredient used; nut-based products were usually lower in saturated fat, whereas coconut-based ones had a higher content compared with dairy cheese. For milk and yoghurts, PB alternatives contained higher total fat and fiber, and lower protein and saturated fat compared with their animal-based counterparts. The amount of sugar in milk, yoghurts, and plant-based alternatives was comparable.
Katidi et al. [46] (2023)	Food label analysis from the Greek Branded Food Composition Database	Cheese (*n =* 172; PB *n =* 80), milk (*n =* 119; PB *n =* 221), and yoghurt (*n =* 137; PB *n =* 40) compared with PB alternatives	Milk and yoghurt alternative products had lower total fat and saturated fat content than their animal counterparts. Plant-based cheese imitations contained higher content of SFA, carbohydrates, and fiber and they were lower in protein, compared with animal-based cheese products. All dairy imitations had lower protein content than animal-based dairy.
Lee et al. [47] (2023)	Food label analysis of products on the Canadian market	Cheese (*n =* 947; PB cheese *n =* 24), yogurt (*n =* 583; PB *n =* 33) and milk (*n =* 279; PB *n =* 150) compared with PB alternatives	Plant-based dairy alternatives had lower protein, but higher carbohydrate, sugar, and fiber content compared with animal products. Plant-based milk had lower energy, total fat, and saturated fat; plant-based yoghurt had lower sodium; and all had lower calcium. Plant-based cheese and yoghurt were more expensive than their animal-based counterparts, but milk prices were comparable.
Moshtaghian et al. [30] (2024)	Food label analysis and Swedish food composition database	Plant-based alternatives to milk (*n =* 73), yoghurt (*n =* 41), cheese (*n =* 36), cream (*n =* 22), ice cream (*n =* 23), and fat spread (*n =* 27) compared with corresponding dairy products (n not reported)	Plant-based dairy alternatives had higher fiber but lower protein than dairy products, except for soy-based options. They had similar or lower saturated fat and energy content, except for coconut-based yoghurt, plant-fat-based cheese, fat spread, and ice cream, which were higher. Fortification with vitamins D, B2, B12, calcium, and iodine is common, matching or exceeding dairy levels, except in plant-based milk. Organic plant-based milk and yoghurt have lower micronutrient content than nonorganic varieties, except for vitamin D.
Pointke and Pawelzik [48] (2022)	Food label analysis based on online European market analysis	Cheese compared with PB alternative (*n =* 65 in 2019; *n =* 123 in 2021)	Plant-based cheese alternatives generally had lower protein and calcium content compared with dairy cheese, but higher carbohydrate and saturated fat levels due to ingredients such as coconut oil. Although these alternatives can meet daily requirements for certain vitamins such as E and K, they lack essential nutrients like Vit B12 unless fortified.
Romão et al. [8] (2022)	Food label analysis of Brazilian products (focused only on macronutrients)	PB "milk"/beverages (*n =* 80), yoghurts (*n =* 11), and cheese (*n =* 45), mayonnaise (*n =* 14) and eggs (=2)	No differences were found regarding the energy value of plant-based and animal versions of all included samples. Only the categories of cheese and eggs presented more carbohydrates than their animal counterparts. Cheese and beverages had more protein than their plant-based alternatives. No differences were found regarding the total fat content of the samples, except the animal samples of mayonnaise and eggs which had greater levels of saturated fats. Plant-based yoghurts showed more fiber than their animal counterparts. Regarding the ingredients, cashew nuts were the most used matrixes in plant-based dairy products, whereas rice and coconut were the most common in the included beverages.

Abbreviations: PBAF, plant-based alternatives to animal food; PB, plant-based; PBDA, plant-based dairy alternatives.

##### Yoghurt

Plant-based yoghurts are most commonly based on soy, almonds, oats, and coconut. Compared with dairy yoghurt, plant-based alternatives generally contain lower protein levels and saturated fat (Table 5), with soy-based varieties being the most comparable to dairy in protein content. Although some products are fortified with essential micronutrients such as calcium and vitamin B12, the rates of fortification vary [43].

#### Egg alternatives

Few studies have assessed plant-based alternatives to eggs; for the present review, 5 studies were found. The PBAF eggs vary largely in their nutritional composition, as seen in Table 6 [6,9,27]. PBAF eggs have a higher carbohydrate content than chicken eggs and much lower cholesterol and saturated fats. Large variations in the PBAFs' total fats and protein content make comparing these products as a group with chicken eggs difficult, with alternatives having both more and less of these nutrients than eggs.

**TABLE 6:** Nutritional composition of chicken egg and plant-based alternatives to eggs, given for 100 g of food product

Reference	Chicken egg	Plant-based egg alternatives								
	McClements and McClements [27] (2023)	Mean ± SD	Romão et al. [8] (2022)	Romão et al. [8] (2022)	McClements and McClements [27] (2023)	McClements and McClements [27] (2023)	McClements and Grossmann [27] (2021)	McClements and Grossmann [27] (2021)	Tso et al. [33] (2021)	de las Heras-Delgado et al. [6] (2023)
Energy (kcal)	143	208 ± 83	250	400	159	170	175	175	183.85	152.4
Carbohydrates (g)	1	9.5 ± 11.7	33	17.1	2	3.8	2.3	5.3	2.65	—
Protein (g)	12.4	17.9 ± 15.5	41	44.3	11	11.3	11.4	12.3	8.8	3.4
Total fats (g)	10	11.9 ± 3.5	8	17.1	11	13.2	11.4	12.3	15.2	6.7
Saturated fats (g)	3.2	0.6 ± 0.7	0	0	0	1.9	0	0.9	0.87	0.8
Monosaturated fats (g)	3.6	8.1 ± 1.5	—	—	—	7.0	—	—	9.1	—
Polyunsaturated fats (g)	1.8	3.5 ± 0.5	—	—	—	3	3.4	3.5	4.3	—
Cholesterol (mg)	411	0.7 ± 1.2	—	—	—	0	0	—	2.1	—
Dietary fiber (g)	—	13.4 ± 0.8	14	12.9	—	—	—	—	—	—
Calcium (mg)	48		—	—	—	—	—	—	24.2	—
Iron (mg)	1.67		—	—	—	3.4	—	—	—	—
Vit B12 (μg)	1.02		—	—	—	—	—	—	—	—
Zinc (mg)	1.24		—	—	—	—	—	—	—	—

## Discussion

### Nutritional quality of PBAF

The nutritional quality of PBAFs varies, depending not only on the animal food they are designed to substitute but also on similar products. PBAFs are generally different from their animal counterparts in terms of nutritional composition. Regarding macronutrients, most PBAFs are lower in total fats and saturated fats (TABLE 1, TABLE 2, TABLE 3, TABLE 4, TABLE 5), except for cheese alternatives. Due to their formulation, many plant-based kinds of cheese do not provide the same nutrients as dairy cheese, requiring further optimization to improve their nutritional profile. Meats and yoghurt alternatives often have a higher content of carbohydrates than their animal counterparts. In comparison, milk alternatives usually only have a higher carbohydrate content than milk if they are based on cereals (i.e., oats and rice). Protein is generally lower in PBAFs, but more prominent differences between plant- and animal-based foods are observed in dairy products (TABLE 4, TABLE 5) and eggs (Table 6).

Animal-based foods generally have a more consistent macronutrient profile within categories—for example, meat and fish [51,52], although there is variability in their nutritional composition as well. This variability can be influenced by factors such as the animal’s environment, feeding strategies, and genetics. For example, the protein, fat, and lactose composition of cow milk varies according to season, feeding strategies [53], and other factors such as genetics [54]. Nevertheless, compared with plant-based alternatives, animal foods tend to exhibit relatively more consistent nutritional profiles. Similarly, the nutritional composition of PBAFs varies significantly. In PBAFs, the variation is driven by differences in formulation, ingredients used, and the fortification status of the products. This is particularly clear in the PBD category (Table 4), where variation is observed among products from different raw materials. This is most evident for protein content, as PBDs made from soy exhibit higher protein levels than other PBDs. Although in PBAFs for meats (Table 2), variability in nutritional composition is explained by a more extensive ingredients list and recipe variability. The micronutrient composition of these products varies and depends heavily on fortification. Many PBAFs are fortified with essential micronutrients such as calcium, vitamin D, and vitamin B12 to match the nutritional profile of dairy milk and meat [30].

The modeling studies assessed in this review (Table 1) found theoretical differences in dietary intake of nutrients when PBAFs are used to replace dairy products, meat products or both. In summary, they have mentioned that there would be a decrease in the intake of protein and SFAs, and an increase in the intake of dietary fiber. As for micronutrients, there would be a higher intake of iron but a decrease in bioavailable iron intake and a decrease in the intake of vitamin B12, riboflavin, iodine, and calcium. Not all studies mention these results, but not necessarily because they observed divergent results, but because some of the nutrients were not assessed or included in the publication.

In 5 of the 10 studies assessing the nutritional composition of meat alternatives, the protein content in the PBAFs was similar to meat. However, the biological quality of this protein must be assessed. Plant proteins have poorer quality, even in isolated forms, as no plant source can provide all essential amino acids (EAAs), requiring a combination of ≥2 raw materials, like a cereal and a legume [55].

Protein quality is explored in one of the modeling studies reviewed [38], which reported a decrease of ≥10% in usable protein in meat replacement with PBAFs. According to the findings of Wanders et al. [38], soy-based meat PBAFs had a Protein Digestibility Corrected Amino Acid Score (PDCAAS) of 1.0, which means that all EAAs are provided in the required amounts. Contrastingly, pea and wheat had PDCAAS values of 0.73 and 0.34, suggesting that each lack ≥1 EAA in the required amounts.

As for dairy, the study of Singh-Povel et al. [31] analyzed the protein quality of cow milk and milk PBAFs. Their analyses compared 1 glass of cow milk, which provides 24% of the WHO EAA requirements, with 1 glass of PBAFs drink and found that PBAFs have significantly lower amounts of EAA. To meet the same amount of EAA provided by a glass of cow milk, one would need to consume 1.7 glasses of soy drink or 246.3 glasses of rice drink [31]. It should be noted that despite the decrease in usable protein and EAA, the study of Wanders et al. [38] found that protein intake was still adequate for 88% of the Dutch adult population. Similarly, a modeling study in Australia also showed that all substitution scenarios (i.e., meat, dairy and meat + dairy), the theoretical intake of protein would be above recommendations, and another study in the Netherlands [20], found that only 3% of the population would have a total protein intake below the recommendations, whereas 19% would have excessive intake. These results showing total protein intake above the recommendations could mean that some of the scenarios the EAA could potentially be adequate, but these analyses were not conducted by the authors [18,20].

Milk and dairy are important sources of iodine in Western diets [56]. The intake of iodine from dairy is even more important in countries where iodine fortification in staple ingredients, such as iodized salt, is not mandatory [49]. Iodine content in PBAFs is often overlooked in nutritional composition studies (Table 4), probably because it is not a nutrient used in the fortification of these products and declaration in nutrition information tables is not mandatory. In modeling studies (Table 1), the substitution of milk and dairy products with PBAFs resulted in an increased risk of iodine deficiency [17,19].

Iodine is used in the synthesis of thyroid hormones, which are important metabolic modulators, and essential for growth and neurodevelopment in the fetal stage and childhood [57]. Iodine deficiency causes hypothyroidism (decreased function of the thyroid), goiter (growth of the thyroid), neurological issues, especially in children, and reproductive issues [58].

Calcium is another mineral whose intake comes mainly from dairy [59]. Plant-based alternatives to milk often seem to be fortified in calcium levels compared with cow milk (see Table 4), whereas alternatives to other dairy products (see Table 5) are often lower in calcium than their animal counterparts. Not all modeling studies assessed in the present article (Table 1) have pointed to calcium as a nutrient of potential deficiency in the total replacement of dairy with PBAFs. Specific population needs and variability in fortification are determinants for risk of calcium deficiency. However, calcium from plant sources might be less bioavailable due to antinutrients that inhibit calcium absorption. Calcium absorption is mainly inhibited by oxalates and, to a less extent, by phytates [59,60]. Therefore, the concentration of antinutritional compounds is particularly important in meals with sources of calcium in individuals who follow plant-rich diets.

Adequate intake of calcium is particularly important for children and adolescents forming their calcium reserve in the bones, as well as females who are pregnant, lactating and postmenopausal [57,61]. Calcium is important for growth and bone health, but it is also important for the function of other tissues, such as muscle contraction, blood clotting, and nervous synapses [57]. Long-term calcium deficiency leads to rickets in children and osteoporosis in adults and can increase risk of high blood pressure and preeclampsia [62].

Vitamin B12 is almost exclusively found in animal foods, with the exception of some fermented plant foods [32]. In this review, a large variability in the content of vitamin B12 was observed (TABLE 2, TABLE 4, TABLE 5); from no fortification to being added to amounts similar to or higher than their animal counterparts. Most of the modeling studies reviewed in Table 1 indicated a decrease in vitamin B12 intake in the PBAF scenarios. The systematic review of Neufingerl and Eilander [63] showed that the intake and status of vitamin B12 are lower in vegetarians and vegans compared with meat eaters, and the prevalence of deficiency is much higher in vegetarians and vegans.

A deficiency of vitamin B12 leads to megaloblastic anemia and, consequently, fatigue due to improper oxygen transport [57,64]. Vitamin B12 is also essential to neurological function, and its deficiency causes cognitive deficits, mood changes, confusion, and depression [65]. Considering a future with plant-richer diets, vitamin B12 fortification of plant-based foods could be a public health strategy to prevent possible deficiencies of this nutrient [32,50,66].

Iron was higher in PBAF than in meats, as mentioned in both modeling studies (Table 1) and in nutritional composition studies **(**TABLE 2, TABLE 3). A higher content and, consequently, intake of iron from plant origin likely does not translate into better iron status. As mentioned in the results of the modeling study of Salomé et al. [19], a decrease of bioavailable iron would be observed with the substitution of meat by PBAF. This result likely applies to the studies that mentioned an increase in iron intake [16,17,20]. Iron naturally occurs in plants in its nonheme form, Fe3+, and it is also the form of iron used to fortify foods [67]. Nonheme iron is less bioavailable than heme iron, which is only present in animal foods [32]. Aligned with this argument are the results of a systematic review by Neufingerl and Eilander [63] that found that vegans had a higher iron intake than vegetarians and meat eaters but also a lower iron status.

In general, iron deficiency leads to a compromised immune system, alopecia, reduced concentration, and dizziness [68]. In pregnancy, iron deficiency in mothers can impair proper fetal growth, low birth weight, and premature labor [69], and in children, it can diminish neurocognitive development [68].

In the modeling studies (Table 1) that analyzed zinc intake (3/8 studies), zinc intake would decrease with the partial or total substitution of meat and milk for PBAFs. Similarly, vegans and vegetarians have a higher risk of inadequate intake of zinc because of the lower bioavailability of zinc from plant foods [63]. There are no good biomarkers of zinc status [70], which can impact the uncertainty of the impact of different diets on zinc status [63]. Severe zinc deficiency is rare in high-income countries and mostly occurs due to nondiet causes, but in low-income countries, it manifests in stunting and frequent infections [70].

Besides the lower bioavailability of nutrients in plant sources, like nonheme iron, plant foods contain phytates and other food components that reduce their bioavailability even further [67]. Moreover, meat eaters benefit from the meat factor. Meat factor is an intrinsic component of animal flesh, and studies have shown that having meat in a meal increases the absorption of zinc and iron from plant foods in the same meal [71].

Riboflavin, or vitamin B2, was mentioned by 2 (of 9) modeling studies as potentially insufficient intake in the PBAF scenarios (Table 1). Riboflavin content was not often highlighted by studies of meat PBAFs (1/10 studies, Table 4) or for dairy and eggs PBAFs (2/11 studies, Table 5). Interestingly, PBDs are often fortified with riboflavin in amounts similar to those of cow milk (Table 4).

Vitamin B2 is found in both animal and plant foods, and intake in meat eaters, vegetarians, and vegans is similar [63]. It works as a precursor of coenzymes involved that are central to cellular respiration and energy generation and in the metabolism of other complex B vitamins and iron [72]. Riboflavin is an important antioxidant in the body, and its deficiency can lead to oxidative stress [73]. Riboflavin deficiency manifests in different systems, including neurological alterations, inflammation and irritation of different tissues and anemia [72].

Low-income families or people living in rural areas are vulnerable to riboflavin deficiency, which led to public policies in different countries of fortifying white flour with riboflavin [74]. Moreover, it has been suggested that riboflavin deficiency caused by a vegan diet in pregnancy can lead to life-threatening neonatal conditions, with 1 case reported [72,75].

Because PBAFs are variable in their nutritional composition (both compared with animal products and other PBAFs), it is important that the consumer is aware of these differences and equipped with the knowledge to choose the most appropriate alternative for them and their dietary needs. Differences in fortification within the same food category can be confusing for consumers and increase the pressure on the consumer to discern how adequately the PBAFs are supplying their nutritional needs. For example, the consumer should be aware that the main source of calcium in their diet is milk and cheese and, therefore, need to find PBAFs with similar calcium content or find this nutrient in other food or supplements.

Going forward toward plant-rich diets, current fortification policies might need to be reviewed, with or without a large intake of PBAFs. People will need to be educated and prepared to make informed choices. Additionally, specific labels indicating if the PBAFs match the nutrient profile of their animal counterpart for key nutrients the animal foods are source of such as vitamin B12 for meats and calcium for dairy.

If one follows a plant-rich diet such as the Planetary Health Diet [1], that includes small intakes of animal foods, the deficiency risk is much lower than if animal foods are to be completely removed from diets. Following a study in the United Kingdom, where salt is not fortified with iodine, it has been suggested that Planetary Health Diet is adequate for adequate intake of iodine, although, if nonfortified PBDs are consumed instead of milk, the population would be in deficiency risk [76].

Considering the predicted growth of consumption of PBAFs, regulations for the formulation of these products could be introduced. Moreover, it is important that the nutritional pitfalls of consuming PBAFs instead of animal foods are clear to consumers so informed choices can be made. Tso et al. [9], have suggested that we have guidelines on the minimum adequacy of protein and micronutrients that would make a food be considered an adequate replacement for animal protein. With guidelines or regulations, more nutritionally adequate foods can be produced while still providing more environmentally friendly alternatives. Mandatory fortification of PBAFs in levels similar to animal foods, for example, could reduce risks of deficiencies caused by replacing animal foods with PBAFs.

Food composition databases need to be updated including these new PBAFs to monitor nutritional status and risk-benefit assessment should focus on dietary changes resulting from the introduction of PBAFs into habitual diets. Risk-benefit analysis using modeling studies could help prevent nutritional trade-offs caused by increased intake of PBAFs.

### Environmental impact of PBAFs

One of the motivators for the increasing offer and consumption of PBAFs is the environmental burden of animal food production. Livestock is responsible for about 14.5% of global greenhouse gases (GHG) emissions from human activities and nearly three-quarters of agricultural land [77]. In contrast, animal foods contribute to only 18% of calories and 25% of protein in the food consumed worldwide [78].

It should be noted that livestock has an important role in biodiversity conservation by grazing and pasture management and can occupy land that has no other use [79]. However, not all livestock depends solely on grazing. Feed production often takes place on arable land that could be used for food production [78]. Moreover, land now occupied by livestock could be used as conservation or reforestation areas [2], particularly in areas where biodiversity was removed for livestock production, such as in the Amazon region. Compared with their animal counterparts, the environmental impact of PBAFs varies depending on the functional unit (FU) used in the comparison. Using the FU of 100 g of protein, meat alternatives emit 10–13 times less GHG emissions than beef and 2–3 times lower emissions than pork and poultry protein [40]. Despite high processing, PBAFs to beef patties have been shown to emit 90% less GHG and use less energy, land, and water to be produced, compared with cattle beef [12].

As for plant-based alternatives to milk, if the FU used is volume (e.g., 1L of milk/PBD), the plant-based alternatives have a much lower environmental impact [80,81]. However, if nutritional components are used as FU, such as protein quantity and quality and nutrient density, in most cases, PBDs require more natural resources for production than cow milk [10]. In a recent study in 6 European countries, only soy drinks had a lower environmental impact than cow milk, having protein quality as FU [31].

### Consumer perceptions

Beyond nutritional adequacy and environmental impact, it is important that consumers substitute their habitual animal food intake for PBAFs. In this context, we need knowledge about consumers’ willingness to change and what the main drivers and barriers to consume PBAFs are. This knowledge can improve PBAFs aspects that consumers find challenging. Several studies have investigated consumers’ willingness to buy and to eat PBAFs [82,83]. Overall, consumers report that PBAFs’ sensory properties (particularly taste) and price are the main barriers to consumption [13].

Regarding price, PBAFs tend to be more expensive than their animal counterparts [84], particularly dairy PBAFs [85,86]. Moreover, consumers perceive the price of PBAFs as too costly for a poorer sensory aspects when compared with other products [87,88].

As for sensory properties, consumers often report that PBAFs are not similar enough to their animal counterparts. Formulating PBAFs that mimic animal foods requires overcoming a series of technological challenges to properly balance a mix of ingredients, whereas animal foods are mainly based on a single food matrix [89]. The mix of ingredients often results in poor sensory properties, such as a rubbery texture in meat PBAFs, a grainy mouthfeel in dairy PBAFs, and a perception of “too artificial” flavors [90,91]. Moreover, consumers who have a habitual intake of meat and dairy tend to have poorer perceptions of PBAFs than vegans and vegetarians [92], as they have animal food as a reference of comparison.

Besides product-related properties, there are consumer-related barriers to the consumption of PBAFs. Notably, the role that meat and dairy consumption play in different societies which are deeply embedded in culture. Western high-income countries often present a high attachment to meat and dairy; that is, an intrinsic positive to consume these products [87,93,94]. Moreover, growth in animal intake in lower-income countries is a social marker of increased wealth and status [95].

Considering these arguments, to increase the popularity of PBAFs and the Great Food Transition, policymakers and the food industry should be aware that PBAFs should not only resemble animal foods but also be superior to them in all aspects: nutrition, environmental impact, price, function, and taste.

### PBAFs in current and future diets

A recent study in the United Kingdom concluded that PBAFs’ role in diets is to substitute animal foods for plant-based alternatives in a “like-for-like,” that is, without compromising taste, function, or cooking skills [5]. This substitution is not one-for-one in families’ budgets. Studies in Europe [31] and North America have shown that PBAFs are usually more expensive than milk, dairy, and meats [31,40,47,96]. Price is reportedly a strong motivator for consumers to purchase and consume PBAFs [83,97], and lower prices could potentially increase PBAFs intake.

In the same context, the increased consumption of PBAFs might translate into a higher intake of ultraprocessed foods (UPFs) [6,98], as PBAFs are often more processed than their animal counterparts. This is due to the need for a combination of different raw materials, culinary ingredients, and maybe additives to formulate a PBAF that mimics the flavor and other sensory characteristics of animal protein. Moreover, higher meat avoidance has been associated with a higher UPF consumption in the French NutriNet-Santé cohort [99].

UPFs have been associated with noncommunicable diseases and malnutrition in several observational studies [100]. Moreover, consumers associate UPFs with unhealthy composition [101] and have reported avoiding consuming UPFs when possible [102], which could be a barrier to PBAF consumption. However, not all UPFs are the same, as shown by epidemiological studies looking into UPF subgroups. Ready-to-eat dishes, meat products, and sweetened beverages are positively associated with type 2 diabetes and morbidity of cancer and cardiometabolic diseases, whereas other UPF subgroups have shown negative associations or no associations with these diseases [100,103,104]. UPF PBAF explicitly has shown no associations with morbidity of cancer and cardiometabolic diseases in the analysis of Cordova et al. [103].

However, there is still a discussion on whether UPF PBAFs are healthy. A recent study compared unprocessed animal products with UPF PBAFs and found that PBAFs consumption may lead to an improvement in biomarkers of cardiovascular disease risk compared with animal food intake [105]. However, UPF PBAFs might be less healthy than less processed plant foods, such as mentioned in the studies of Farsi et al. [16], and Tso and Forde [33]. PBAFs could be part of the Great Food Transformation, but not in total replacement of the current intake of animal foods. In their study, Tso and Forde (2021) argued that the current focus is on replacing animal foods with fast foods, whereas it should be on increasing “nutrient-dense vegetable-based foods.” In fact, sustainable food patterns should rely on minimally processed foods that could be reproduced at home, a shift from having animal protein as the protagonist of the meal to plant-rich balanced meals.

### Limitations

The main limitation of this review is the mix of methods used to assess the nutritional composition of PBAFs. Most studies have sourced their data from the products’ labels. Food labels are often based on calculations rather than chemical analysis and can therefore be imprecise [106]. One of the studies in this review is a study on milk PBAFs in Italy that showed that most labels provided imprecise information [34].

Moreover, most micronutrients are not assessed because their content is not required to be reported in food labels. A few studies evaluated in this review have focused on micronutrients and have performed chemical analysis to determine nutrient content, but have also been limited to a few micronutrients, such as iodine in dairy and PBAFs in the study of Alzahrani et al. [49] (Table 5 and Supplemental materials).

Another limitation of our review is the focus on nutritional quality. As foods and diets are not limited to nutrition, a holistic approach that includes social, cultural, environmental, and health aspects is needed to properly assess whether a food group is adequate for inclusion in diets. Although we briefly discussed these topics, a deeper transdisciplinary assessment is needed.

Moreover, most studies available on PBAFs are conducted in high-income countries, mainly in North America, Europe, and Australia. Therefore, the current knowledge on PBAFs is limited to countries with similar backgrounds and dietary patterns, whereas other food cultures that have traditional use of PBAFs are not represented.

In conclusions, it is noted from the present review that PBAFs have a role in easing the transition toward plant-rich diets, the Great Food Transformation. However, the complete substitution of the current intake of animal foods for PBAFs available on the market today is not recommended as it would put populations at risk of nutritional deficiencies. Because the nutritional composition of PBAF is variable and highly dependent on the raw ingredients used and the level of fortification, these factors should be considered by consumers seeking nutritionally balanced alternatives.

The increased consumption of PBAFs should be considered in health policies, such as Food-Based Dietary Guidelines and compulsory enrichment of food products with micronutrients, such as iodine fortification in salt. As PBAFs are mostly industrially designed, there is a window of opportunity to formulate new products with better nutritional composition that address different needs.

Conversely, these products can potentially be part of new plant-rich dietary patterns by offering familiar tastes and dishes. Concurrently, a plant-rich dietary pattern will require the development of new tastes and preferences, where novel plant-based products could provide nutritious and delicious foods without the need for resemblance to animal foods.

## Author contributions

The authors’ responsibilities were as follows – BPR, SGB: conceptualization and methodology; BVA, DVB, SGB: funding acquisition, supervision and writing – review & editing; BPR: project administration; BPR, LO: data curation, formal analysis and writing – original draft.

## Data availability

Data collected from nutritional composition studies and databases on plant-based alternatives to meats, milk and eggs are available as supplementary materials.

## Declaration of generative AI and AI-assisted technologies in the writing process

During the preparation of this work, the authors used Grammarly to correct grammar and improve the readability of the text. After using this tool/service, the authors reviewed and edited the content as needed and take full responsibility for the content of the published article.

## Funding

This research is part of a PhD project funded by the Sino-Danish university partnership, the Sino-Danish Centre.

## Conflict of interest

BPR reports financial support was provided by Sino-Danish Centre for Education and Research. The other authors declare that they have no known competing financial interests or personal relationships that could have appeared to influence the work reported in this article.
